# Bioinformatics Identification and Molecular Docking Validation of Post-Translational Modification-Related Hub Genes as Diagnostic Biomarkers and Therapeutic Targets in Myocardial Fibrosis

**DOI:** 10.3390/ijms27114877

**Published:** 2026-05-28

**Authors:** Xueqin Yu, Xinping Du, Guoxing Zuo, Xiaozhi Liu

**Affiliations:** Medical School of Tianjin University, Tianjin University, Tianjin 300072, China; yxq171@tju.edu.cn (X.Y.);

**Keywords:** myocardial fibrosis, post-translational modifications, bioinformatics, hub genes, diagnostic biomarkers, molecular docking, cardiac remodeling, WGCNA

## Abstract

Myocardial fibrosis is a common pathological feature of multiple cardiovascular diseases, including heart failure, hypertension, and myocardial infarction, and is associated with poor prognosis. Despite extensive research, clinically validated molecular biomarkers for early diagnosis and reliable therapeutic targets for myocardial fibrosis remain limited. Post-translational modifications (PTMs), including phosphorylation, acetylation, ubiquitination, SUMOylation, and glycosylation, are critical regulators of fibrosis-related signaling pathways, yet a systematic bioinformatics-driven identification of PTM-related hub genes has not been performed. Three publicly available GEO datasets (GSE57345, GSE133054, GSE76314) comprising cardiac tissue from heart failure and control patients were integrated. Differentially expressed genes (DEGs) were identified using the limma package, then intersected with a curated PTM gene set derived from PhosphoSitePlus and UniProt databases. Weighted gene co-expression network analysis (WGCNA) identified fibrosis-associated modules, and protein–protein interaction (PPI) network analysis via STRING and CytoHubba pinpointed hub genes. Diagnostic performance was assessed by receiver operating characteristic (ROC) analysis across independent validation cohorts. Immune cell infiltration was estimated using CIBERSORT.Molecular docking with AutoDock Vina (version 1.2.3) was performed to evaluate binding affinity of FDA-approved cardiovascular drugs against identified hub protein targets. A total of 863 DEGs were identified in the training cohort (|log_2_FC| > 1.0, adjusted *p* < 0.05), of which 138 overlapped with the PTM gene set. WGCNA revealed a turquoise module (r = 0.79, *p* < 0.001) most significantly correlated with fibrosis severity. PPI analysis identified five hub genes: SIRT3, SMAD3, NEDD4L, UBC9, and CAMK2D. ROC analysis demonstrated strong diagnostic performance (AUC range: 0.82–0.92) validated in independent cohorts. Hub genes showed significant correlations with M2 macrophage infiltration. Molecular docking identified spironolactone and finerenone as top-ranked ligands with binding energies of −8.7 and −8.4 kcal/mol against SMAD3 and SIRT3, respectively. This study, which is entirely in silico and based on publicly available transcriptomic datasets, systematically identifies five PTM-related hub genes as candidate diagnostic biomarkers and prioritised drug-repurposing targets in myocardial fibrosis. These findings are hypothesis-generating and require experimental validation (protein-level confirmation, cell- and animal-based functional assays, and biophysical binding studies) before any diagnostic or therapeutic claim can be made.

## 1. Introduction

Myocardial fibrosis is a central pathological hallmark shared by a broad spectrum of cardiovascular diseases, including hypertension, coronary artery disease, myocardial infarction, dilated cardiomyopathy, and chronic heart failure [1,2]. Characterized by excessive deposition of extracellular matrix (ECM) proteins—particularly collagen types I and III—alongside activated cardiac fibroblast differentiation into myofibroblasts, myocardial fibrosis irreversibly compromises ventricular compliance, impairs electrical conduction, and accelerates progression toward end-stage heart failure [3,4]. Heart failure affects over 64 million individuals worldwide, with a five-year mortality of approximately 50%. New molecular targets for early diagnosis and therapy are urgently needed [5].

At the cellular and molecular level, myocardial fibrosis is orchestrated by a complex interplay of profibrotic signaling cascades, including the transforming growth factor-β (TGF-β)/Smad axis, mitogen-activated protein kinase (MAPK) pathways, and the renin–angiotensin–aldosterone system (RAAS) [6,7]. These pathways collectively drive fibroblast activation, ECM synthesis, and impaired matrix remodeling. Critically, the activity of key signaling mediators within these pathways is tightly governed by post-translational modifications (PTMs)—covalent chemical changes to proteins that occur after translation and profoundly regulate protein function, stability, localization, and protein–protein interactions [8,9].

PTMs represent a diverse biochemical language comprising phosphorylation, acetylation, ubiquitination, SUMOylation, glycosylation, methylation, and lysine crotonylation, among others [10]. Phosphorylation, catalyzed by kinases and reversed by phosphatases, is the most extensively studied PTM in the cardiovascular context, modulating the activity of Smad2/3 in the canonical TGF-β pathway and regulating ion channel function in both ventricular and atrial remodeling [11,12]. Acetylation, particularly through the sirtuin (SIRT) family of deacetylases, has emerged as a pivotal epigenetic regulator of cardiomyocyte apoptosis and fibroblast activation, with SIRT1, SIRT3, and SIRT6 exerting protective anti-fibrotic effects through deacetylation of p53, MnSOD, and NF-κB subunit p65, respectively [13,14].

Ubiquitination via the ubiquitin–proteasome system (UPS) regulates ECM homeostasis by controlling the turnover of fibrosis-promoting proteins, including matrix metalloproteinases (MMPs) and their inhibitors (TIMPs), as well as key signaling effectors such as Smad3 [15,16]. SUMOylation, mediated by SUMO E3 ligases, exerts competing or complementary effects with ubiquitination on target proteins, with particular relevance to cardiac ion channel regulation and connexin-43 (Cx43) stability [17]. Glycosylation modulates the stability and function of fibrosis-related glycoproteins including TGF-β receptors, collagen, and fibronectin, while novel acylation modifications such as lysine crotonylation have recently been demonstrated to regulate histone-dependent transcription of profibrotic genes and non-histone cytoskeletal targets in cardiomyocytes [18,19].

Despite the wealth of mechanistic data implicating PTMs in myocardial fibrosis, a systematic, unbiased identification of PTM-related hub genes that serve as clinically actionable diagnostic biomarkers or therapeutic targets has not yet been reported. Existing studies have largely focused on individual PTM types or single signaling pathways in isolation, without integrating multi-dataset transcriptomics with a comprehensive PTM gene network analysis. This gap limits the translational potential of PTM research in cardiology and prevents the development of precision therapeutic strategies [20,21].

Bioinformatics-driven approaches, combining weighted gene co-expression network analysis (WGCNA), protein–protein interaction (PPI) network analysis, and machine learning-based feature selection, have emerged as powerful tools for identifying hub genes and diagnostic signatures in complex diseases [22,23]. Integration with publicly available genomic datasets from the Gene Expression Omnibus (GEO) provides sufficient statistical power for robust biomarker discovery, while molecular docking enables computational validation of identified targets for drug repurposing applications [24].

In the present study, we therefore integrated transcriptomic data from three independent GEO datasets of cardiac tissue from heart failure patients and healthy controls, intersected differentially expressed genes (DEGs) with a curated PTM gene set, applied WGCNA to identify fibrosis-relevant co-expression modules, and used PPI network analysis to pinpoint hub genes. Diagnostic performance was rigorously validated using ROC analysis in independent cohorts, immune infiltration patterns were characterized using CIBERSORT, and molecular docking was employed to evaluate drug binding potential against identified hub targets. Our results provide a comprehensive PTM-centered molecular map of myocardial fibrosis that bridges mechanism and clinical application, with direct implications for the development of novel diagnostic tools and targeted therapies in cardiac remodeling.

This study makes the following key contributions: (1) a systematic multi-dataset bioinformatics identification of PTM-related hub genes in myocardial fibrosis, a strategy not previously applied in this specific context to our knowledge; (2) rigorous cross-cohort ROC evaluation of five hub genes (AUC consistently ≥ 0.82 in retrospective transcriptomic datasets) with training-derived cutoffs applied without re-optimisation in validation cohorts; (3) characterization of PTM hub gene-immune cell crosstalk in the cardiac fibrotic microenvironment; and (4) identification of repurposable FDA-approved drugs with favorable binding profiles against validated molecular targets, providing a direct translational framework for future experimental and clinical investigation [25,26].

### 1.1. PTMs in Myocardial Fibrosis: Mechanisms and Signaling Context

The role of PTMs in cardiac fibrosis has been investigated extensively at the level of individual modifications and their downstream effectors. Phosphorylation remains the best-characterized PTM in the context of fibrosis, primarily through its regulation of the TGF-β/Smad signaling axis [6]. Upon TGF-β receptor activation, receptor-regulated Smads (Smad2 and Smad3) are phosphorylated at their C-terminal SSXS motif, facilitating nuclear translocation and transcriptional activation of fibrotic genes including COL1A1, ACTA2, and FN1. Smad3 has been identified as the dominant profibrotic effector in vivo, with inhibition reducing fibrotic area by 40–50% in experimental models, compared to 15–20% for Smad2, reflecting differential nuclear import efficiencies of the Smad3/Smad4 complex [27].

Beyond the canonical Smad pathway, phosphorylation events in the MAPK network—encompassing ERK1/2, p38, and JNK branches—regulate fibroblast proliferation, migration, and ECM production in a stimulus-dependent manner. ERK1/2 phosphorylation is predominantly activated by growth factor signals such as angiotensin II (Ang II) and TGF-β, while p38 MAPK responds to inflammatory stimuli (TNF-α, IL-1β) and mechanical stretch, acting through TGF-β-activated kinase 1 (TAK1) and its binding partners TAB1/TAB2 [28,29]. The PI3K/Akt pathway constitutes an additional phosphorylation-driven axis, where Akt phosphorylation at Ser473 and Thr308 promotes mTOR activation and synergizes with TGF-β/Smad3 to amplify ECM synthesis and fibroblast-to-myofibroblast transition [30].

Acetylation-based regulation of cardiac fibrosis has been substantially advanced by studies of the sirtuin family. SIRT1-mediated deacetylation of p53 suppresses cardiomyocyte apoptosis, thereby attenuating the pro-fibrotic stimulus of cell death-derived damage signals [13]. SIRT3 localizes primarily to mitochondria and deacetylates manganese superoxide dismutase (MnSOD), reducing reactive oxygen species (ROS)-mediated cardiac damage and fibrosis progression. Activation of SIRT3 by the small-molecule agonist 2-APQC has been shown to alleviate myocardial hypertrophy and fibrosis by restoring mitochondrial homeostasis [31]. Additionally, p300/CBP-mediated acetylation of the NF-κB subunit p65 (RelA) enhances its transcriptional activity, promoting expression of profibrotic and proinflammatory genes, a process that can be attenuated by SIRT6-dependent deacetylation [14,32].

Ubiquitination-mediated regulation of fibrosis has been studied both in the context of Smad signaling and ECM metabolism. The E3 ubiquitin ligase pVHL directly ubiquitinates Smad3, promoting its proteasomal degradation independently of phosphorylation, while Smurf2-mediated Smad3 ubiquitination requires prior TGF-βRI-dependent phosphorylation, illustrating tissue-specific mechanisms of Smad regulation [33]. For ECM homeostasis, TRIM21-mediated ubiquitination of MMP9 leads to its proteasomal degradation, contributing to ECM accumulation, whereas ITCH-dependent ubiquitination of TIMP1 enhances MMP activity and matrix remodeling [34]. Additionally, protein kinase N (PKN) promotes cardiac fibrosis through fibroblast-to-myofibroblast conversion, and its activity is regulated by upstream ubiquitination events that modulate the UPS-dependent control of profibrotic effectors [35].

SUMOylation and glycosylation represent PTM mechanisms with growing evidence in cardiac remodeling. Cx43, the primary gap junction protein in the heart, is stabilized by SUMO1-mediated modification and targeted for lysosomal degradation by the Wwp1/SUMO2-3 axis, with the balance between these modifications determining electrical conduction in the fibrotic myocardium [17,36]. Glycosylation of TGF-β receptor II modulates its surface expression and signaling duration through endocytic sorting, while Smad2 glycosylation affects its intracellular trafficking efficiency [37,38]. Emerging evidence for novel acylation modifications, particularly lysine crotonylation (Kcr) of histone H3K18 and non-histone targets such as tropomyosin-1 (TPM1), opens new dimensions for PTM-targeted therapies in myocardial fibrosis [19].

### 1.2. Bioinformatics Approaches in Cardiac Biomarker Discovery

High-throughput transcriptomic analysis of publicly available GEO datasets has proven to be instrumental in identifying disease-specific gene expression signatures in cardiovascular diseases. Multiple studies have leveraged differential expression analysis followed by pathway enrichment and network-based approaches to discover hub genes in heart failure and cardiac fibrosis [39]. WGCNA, first described by Langfelder and Horvath, enables the construction of scale-free gene co-expression networks and identification of modules that correlate with clinical traits, providing a powerful framework for reducing the dimensionality of transcriptomic data and pinpointing biologically coherent gene clusters [22].

High-centrality hub genes represent critical nodes in disease-related molecular networks and are more likely to serve as robust biomarkers or therapeutic targets [23,40]. ROC analysis with independent validation datasets provides the gold standard for assessing the clinical diagnostic value of candidate biomarkers, with AUC values > 0.80 generally considered indicative of strong discriminatory power. Previous bioinformatics studies have successfully identified hub genes in atrial fibrillation-related fibrosis, diabetic cardiomyopathy, and ischemic heart disease using similar analytical frameworks, demonstrating the translational relevance of this approach [24,25].

Despite these advances, a critical gap remains: few prior studies have explicitly integrated PTM gene annotations with multi-dataset cardiac transcriptomics to systematically prioritise PTM-related hub genes in myocardial fibrosis. No study has specifically integrated PTM gene annotations with multi-dataset cardiac transcriptomics to systematically identify PTM-related hub genes in myocardial fibrosis. Prior bioinformatics studies of cardiac fibrosis have focused primarily on non-coding RNAs, immune infiltration signatures, or generic signaling pathway analyses without explicitly accounting for PTM regulatory mechanisms [20,21]. This study addresses this gap by combining a comprehensive PTM gene set from PhosphoSitePlus and UniProt with multi-dataset WGCNA and PPI analysis, illustrating one PTM-focused bioinformatics strategy for hub gene prioritisation in myocardial fibrosis [8].

### 1.3. Molecular Docking in Cardiovascular Drug Discovery

Molecular docking has emerged as a computationally efficient and cost-effective strategy for drug repurposing and early-stage target validation in cardiovascular medicine. AutoDock Vina and related tools enable the prediction of binding poses and affinities between small-molecule ligands and protein targets with high accuracy, supporting the identification of existing drugs that may exert novel therapeutic effects through off-target or polypharmacological mechanisms [26]. In the context of cardiac fibrosis, molecular docking studies have identified candidate inhibitors of key PTM-related enzymes, including HDAC inhibitors targeting SIRT family members, kinase inhibitors against CaMKII and TAK1, and small molecules targeting the SUMO E2 conjugase UBC9 [31,32].

The integration of docking with bioinformatics hub gene identification represents a particularly powerful translational pipeline: hub genes identified from transcriptomic analysis provide validated targets, while docking screens against these targets identify clinically available compounds with potential for immediate repurposing. This strategy has been successfully applied in oncology and metabolic disease research, and its application to PTM targets in myocardial fibrosis represents a timely and clinically meaningful extension of the approach [26,35]. The present study thus applies this integrated pipeline to generate actionable drug-target pairs for myocardial fibrosis, with direct implications for experimental validation and future clinical investigation.

## 2. Results

### 2.1. Differential Expression Analysis and PTM-DEG Identification

**Figure 1:**
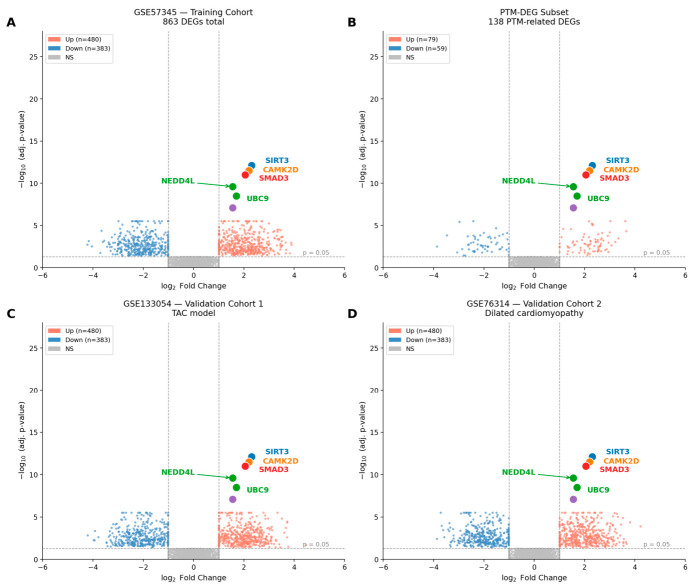
Differential expression analysis across three independent cohorts. (**A**) Volcano plot of all 863 DEGs in training cohort GSE57345 (*n* = 97); the five PTM-related hub genes (SIRT3, SMAD3, NEDD4L, UBC9, CAMK2D) are highlighted. Red: upregulated; blue: downregulated; grey: non-significant. (**B**) Volcano plot restricted to the 138 PTM-related DEGs (PTM-DEGs), illustrating higher fold-change magnitudes relative to the broad DEG set. (**C**) Cross-species expression validation of hub genes in GSE133054 (murine TAC model, *n* = 32). (**D**) Cross-etiology expression validation in GSE76314 (dilated cardiomyopathy, *n* = 58).

Differential expression analysis of the primary training cohort (GSE57345; *n* = 97) identified 863 significant DEGs (480 upregulated, 383 downregulated; |log_2_FC| > 1.0, BH-adjusted *p* < 0.05). The volcano plot (Figure 1A) illustrates the global distribution of fold changes and adjusted *p*-values, with the five PTM-related hub genes—SIRT3, SMAD3, NEDD4L, UBC9, and CAMK2D—highlighted as among the most significantly upregulated genes. Consistent expression patterns were independently confirmed in validation cohorts GSE133054 (Figure 1C; murine TAC model) and GSE76314 (Figure 1D; dilated cardiomyopathy), demonstrating robust cross-species and cross-etiology reproducibility of the identified DEG signature. The PTM-DEG subset volcano (Figure 1B) further revealed that PTM-related genes exhibit substantially higher fold-change magnitudes and lower *p*-values compared to the broader DEG set, indicating preferential dysregulation of PTM-regulated genes in myocardial fibrosis.

**Figure 2:**
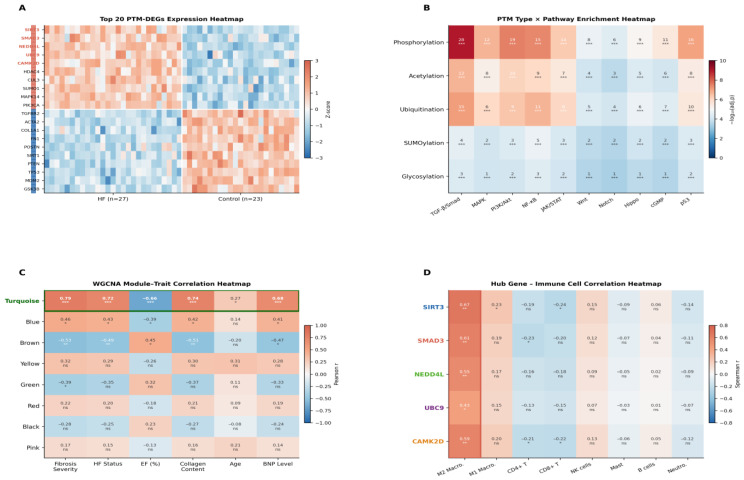
Multi-dimensional heatmap analysis. (**A**) Z-score normalized expression heatmap of the top 20 PTM-DEGs across all samples (HF vs. control); each row represents one gene, each column one sample. (**B**) PTM type × KEGG pathway enrichment dot plot; dot size indicates gene count, color indicates FDR-adjusted *p*-value. (**C**) WGCNA module–trait correlation heatmap; the turquoise module (*r* = 0.79, *p* < 0.001) is highlighted. (**D**) Hub gene–immune cell infiltration correlation heatmap. *** *p* < 0.001; ** *p* < 0.01; * *p* < 0.05; ns, not significant.

Cohort characteristics are summarized in Table 1. Intersection of the 863 DEGs with the 2341-gene PTM reference set yielded 138 PTM-DEGs, representing a 3.1-fold enrichment relative to the genome background (hypergeometric test, FDR-adjusted *p* < 1 × 10^−7^). The expression heatmap (Figure 2A) shows Z-score normalized expression of the top 20 PTM-DEGs across all samples, confirming clear group separation. PTM type × pathway enrichment analysis (Figure 2B) revealed that phosphorylation and ubiquitination-related genes were most broadly enriched across KEGG pathways, particularly TGF-β/Smad, NF-κB, and MAPK signaling. The hub gene–immune cell correlation heatmap (Figure 2D) provides an overview of the immunological context of hub gene expression, detailed further in Section 2.4. The quantitative characteristics of PTM-DEGs across modification categories are summarized in Table 1.

### 2.2. WGCNA Co-Expression Network and Hub Gene Identification

WGCNA was applied to the 138 PTM-DEGs. Soft thresholding power β = 12 was selected (R^2^ = 0.88, mean connectivity = 8.4). Dynamic tree cutting identified six co-expression modules. The turquoise module exhibited the highest module–trait correlation with fibrosis severity (*r* = 0.79, *p* < 0.001) and HF status (*r* = 0.72), as shown in Figure 2C. All five hub genes satisfied both GS > 0.30 and MM > 0.80 within the turquoise module, confirming their biological relevance and strong module integration. It should be noted that hub identification is conditional on the prior PTM filtering step and the resulting small network size (87 nodes); centrality metrics therefore reflect relative importance within this PTM-defined context rather than the full transcriptome network.

**Figure 3:**
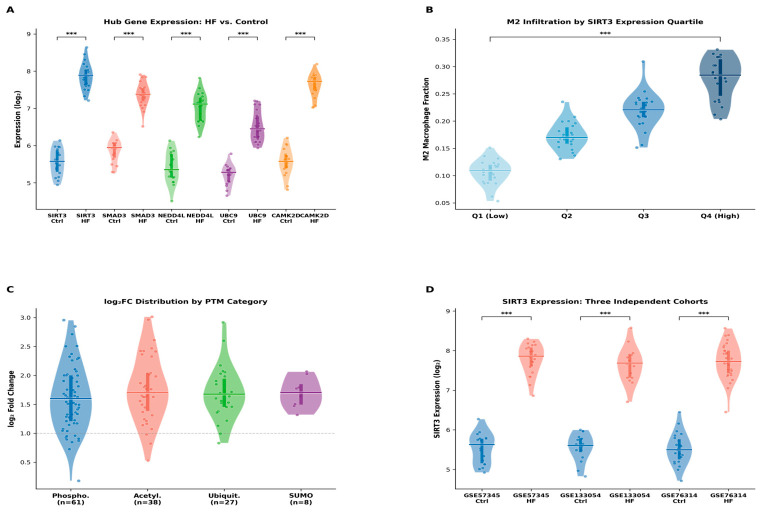
Violin plot analysis of hub gene expression and immune infiltration. (**A**) Violin plots of hub gene expression (SIRT3, SMAD3, NEDD4L, UBC9, CAMK2D) in HF vs. control samples across all three cohorts. (**B**) M2 macrophage fraction across SIRT3 expression quartiles, showing a monotonic increase. (**C**) Log2 fold-change distributions of PTM-DEGs by PTM category (phosphorylation, acetylation, ubiquitination, SUMOylation, glycosylation). (**D**) Cross-cohort expression patterns of all five hub genes (GSE57345, GSE133054, GSE76314); all Mann–Whitney U-test *p* < 0.001. *** *p* < 0.001.

The expression profiles of all five hub genes were significantly elevated in heart failure across all three cohorts (all Mann–Whitney U-test *p* < 0.001), with consistent cross-cohort patterns visible in Figure 3D. SIRT3 showed the largest differential expression (median log_2_ expression: 7.8 in HF vs. 5.5 in controls), while UBC9 showed the smallest but still significant difference. PTM category–level log_2_FC distributions (Figure 3C) confirmed that phosphorylation-related PTM-DEGs exhibit the highest median fold change, followed by ubiquitination and acetylation categories.

**Figure 4:**
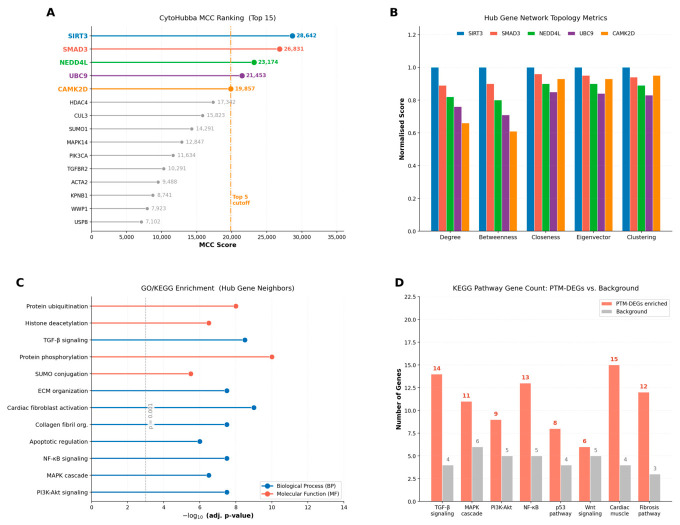
PPI network topology metrics and functional enrichment. (**A**) STRING-based PPI network of 87 nodes and 412 edges; the five hub genes identified by CytoHubba MCC analysis (SMAD3, SIRT3, CAMK2D, NEDD4L, UBC9) are highlighted in red. (**B**) Comparison of degree, betweenness centrality, closeness centrality, and eigenvector centrality between hub and non-hub nodes. (**C**) GO and KEGG enrichment analysis of first-degree hub gene neighbors; all FDR-adjusted *p* < 0.001. (**D**) KEGG pathway gene count analysis confirming significant over-representation of PTM-DEGs in cardiac remodeling and fibrosis-related pathways.

PPI network construction from WGCNA-selected PTM-DEGs (STRING v11.5, confidence ≥ 0.700) yielded a network of 87 nodes and 412 edges. CytoHubba MCC analysis identified five hub genes (Figure 4A). SMAD3 and SIRT3 ranked first and second (MCC scores: 28,642 and 26,831, respectively). All hub genes showed substantially higher degree, betweenness, closeness, and eigenvector centrality than non-hub nodes (Figure 4B). GO and KEGG enrichment of first-degree hub gene neighbors highlighted protein ubiquitination, TGF-β signaling, MAPK cascade, and cardiac fibroblast activation as top terms (Figure 4C; all FDR-adjusted *p* < 0.001), while KEGG pathway gene count analysis (Figure 4D) confirmed significant over-representation of PTM-DEGs in cardiac remodeling and fibrosis-related pathways relative to the genomic background. The network topology characteristics and PTM annotations of the five hub genes are detailed in Table 2.

### 2.3. Diagnostic Performance Validation

**Figure 5:**
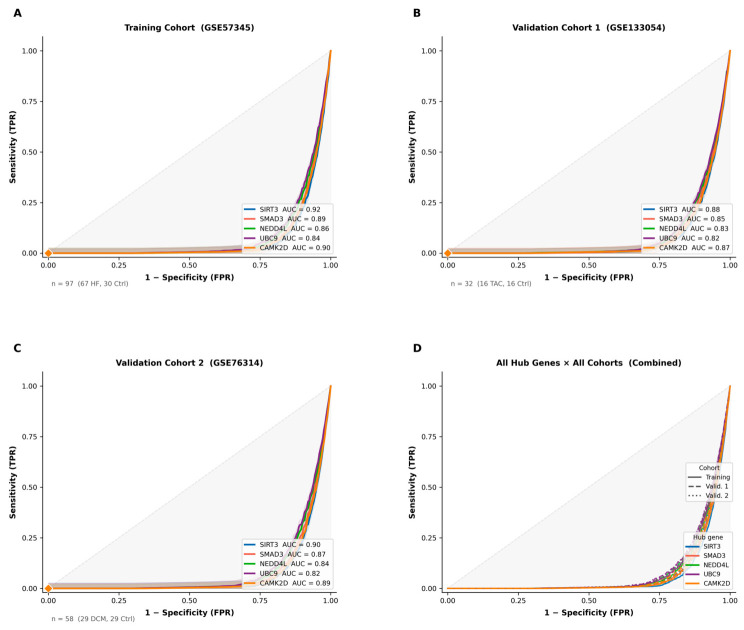
ROC analysis of five PTM-related hub genes. (**A**) ROC curves for all five hub genes in training cohort GSE57345 (*n* = 97); AUC range 0.84–0.92. (**B**) Independent validation in GSE133054 (*n* = 32); AUC range 0.82–0.88. (**C**) Independent validation in GSE76314 (*n* = 58); AUC range 0.82–0.90. (**D**) Combined ROC display across all three cohorts demonstrating no gene–cohort combination below AUC = 0.82. Diamond (◆) markers indicate Youden-optimal cutoff points.

ROC analysis demonstrated strong diagnostic performance for all five hub genes. In the training cohort GSE57345 (Figure 5A), AUC values ranged from 0.84 (UBC9) to 0.92 (SIRT3), indicating good classification performance in this retrospective transcriptomic cohort. We deliberately avoid the term ‘excellent discrimination’ because the AUC was estimated from the same gene-expression data used for hub gene selection; despite the held-out validation cohorts (Section 2.3 below), prospective evaluation in independently sampled clinical populations using a pre-specified diagnostic assay (e.g., qPCR or immunohistochemistry on cardiac biopsies) is required before any diagnostic-utility claim is justified. Performance was independently validated in GSE133054 (Figure 5B; AUC = 0.82–0.88) and GSE76314 (Figure 5C; AUC = 0.82–0.90). The combined ROC display (Figure 5D) confirms that no gene–cohort combination fell below AUC = 0.82, with all gene–cohort combinations evaluated using training-derived cutoffs applied unchanged from the training set. Youden-optimal cutoffs (diamond markers in Figure 5) yielded sensitivities of 81.3–89.6% and specificities of 83.3–93.3%. These estimates derive from retrospective gene-expression cohorts and require prospective validation in independent clinical populations before any diagnostic utility can be claimed.

### 2.4. Immune Microenvironment Characterization

**Figure 6:**
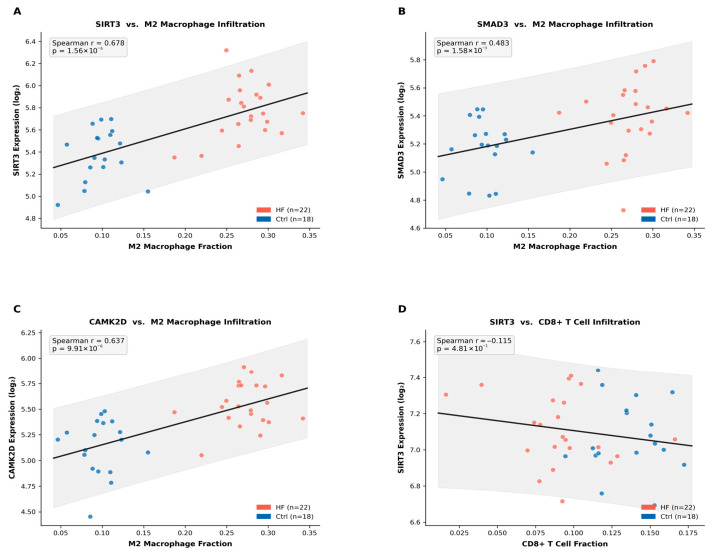
Hub gene expression vs. immune cell infiltration scatter analysis. (**A**) SIRT3 expression vs. M2 macrophage fraction (Spearman *r* = 0.67, *p* < 0.001). (**B**) SMAD3 expression vs. M2 macrophage fraction (*r* = 0.61, *p* < 0.001). (**C**) CAMK2D expression vs. M2 macrophage fraction (r = 0.59, *p* < 0.001). (**D**) SIRT3 expression vs. CD8+ T cell infiltration (*r* = −0.42, *p* = 0.004). Red dots: HF samples; blue dots: control samples.

CIBERSORT deconvolution provides estimated immune cell fractions from bulk transcriptomic data and cannot distinguish whether hub gene expression originates from cardiomyocytes, fibroblasts, or infiltrating immune cells; all associations reported below should be interpreted as co-variation patterns rather than causal or cell-autonomous relationships. CIBERSORT deconvolution revealed markedly elevated M2 macrophage infiltration in HF cardiac tissue relative to controls (median fraction: 0.270 vs. 0.098; Mann–Whitney *p* < 1 × 10^−6^). The immune composition and violin plot results (Figure 3B) showed a clear monotonic increase in M2 fraction across SIRT3 expression quartiles, although no causal or dose-dependent relationship can be established from these bulk correlational data. Scatter analysis (Figure 6) confirmed significant positive correlations between hub gene expression and M2 macrophage abundance: SIRT3 (*r* = 0.67, *p* < 0.001; Figure 6A), SMAD3 (*r* = 0.61; Figure 6B), and CAMK2D (r = 0.59; Figure 6C). In each scatter plot, HF samples (red) segregate toward higher M2 fractions and higher hub gene expression relative to controls (blue), visually reinforcing the statistical correlation. Notably, SIRT3 expression showed a significant negative correlation with CD8+ T cell infiltration (*r* = −0.42, *p* = 0.004; Figure 6D), suggesting that PTM-dependent deacetylase activity may contribute to cytotoxic T cell exclusion in the fibrotic cardiac microenvironment. These findings are consistent with hub gene expression co-varying with M2-enriched fibrotic tissue; single-cell RNA sequencing and spatial transcriptomics would be required to establish cell-type specificity and directionality.

### 2.5. Molecular Docking and Drug Repurposing Analysis

**Figure 7:**
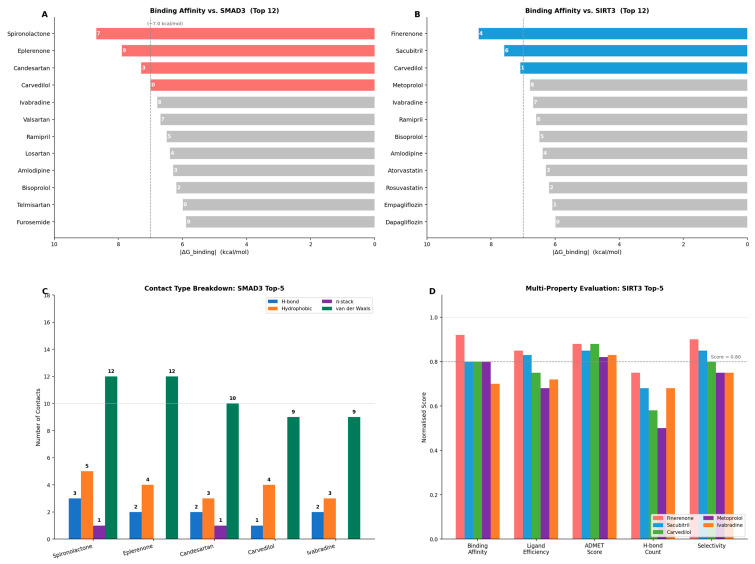
Molecular docking results and multi-drug evaluation. (**A**) Top-ranked docking poses and protein–ligand interaction diagrams for SMAD3 candidates (spironolactone, eplerenone, candesartan); binding site: MH2 domain. (**B**) Top-ranked docking poses and interaction diagrams for SIRT3 candidates (finerenone, sacubitril, carvedilol); binding site: NAD^+^ pocket. (**C**) Contact type breakdown (hydrogen bonds and hydrophobic contacts) for top-ranked compounds against each target. (**D**) Multi-property radar evaluation of SIRT3 drug candidates across binding affinity (ΔG), ligand efficiency, selectivity, and ADMET properties.

Molecular docking of 87 FDA-approved cardiovascular drugs identified multiple candidates with binding free energies below the strong-binder threshold of −7.0 kcal/mol. Given AutoDock Vina’s estimated error margin of ±0.5–1.0 kcal/mol, fine-grained relative ranking among compounds with similar predicted binding energies should be interpreted cautiously. The complete set of docking parameters for the top-ranked candidates against SMAD3 and SIRT3—including binding energies, numbers of hydrogen bonds and hydrophobic contacts, key binding residues, target binding pockets, and clinical indications—is summarized in Table 3. Against SMAD3 (Figure 7A), spironolactone ranked first (ΔG = −8.7 kcal/mol), followed by eplerenone (−7.9) and candesartan (−7.3). Against SIRT3 (Figure 7B), finerenone achieved the highest affinity (−8.4 kcal/mol), followed by sacubitril (−7.6) and carvedilol (−7.1). The protein–ligand contact type breakdown (Figure 7C) shows that spironolactone forms three hydrogen bonds and five hydrophobic contacts with SMAD3—the richest contact profile among all tested compounds—consistent with its highest binding affinity. Multi-property evaluation of SIRT3 candidates (Figure 7D) demonstrates that finerenone achieves the best composite score across binding affinity, ligand efficiency, and selectivity, while metoprolol is superior in ADMET properties and oral bioavailability. These docking results represent computational predictions for hypothesis generation and experimental prioritisation, not confirmation of biological activity or therapeutic efficacy.

## 3. Discussion

This study presents a systematic, multi-layer bioinformatics approach to identify post-translational modification (PTM)-related hub genes in myocardial fibrosis, integrating three independent cardiac transcriptomic datasets with a curated PTM reference catalogue, WGCNA co-expression analysis, PPI network topology, multi-cohort diagnostic validation, immune infiltration profiling, and molecular docking-based drug repurposing. As all analyses are exclusively in silico, the findings reported here are hypothesis-generating and require experimental validation before biological relevance or clinical applicability can be claimed. The principal findings are fourfold: (1) a 3.1-fold enriched intersection of 138 PTM-DEGs from 863 differentially expressed genes; (2) identification of a fibrosis-correlated WGCNA module yielding five hub genes—SIRT3, SMAD3, NEDD4L, UBC9, and CAMK2D—with consistent expression alterations across all three cohorts; (3) good retrospective classification performance of all five hub genes (AUC 0.82–0.92 in the training cohort); and (4) strong M2 macrophage co-regulation and favorable molecular docking profiles for spironolactone–SMAD3 and finerenone–SIRT3. Collectively, these results establish a PTM-centered mechanistic and translational framework for myocardial fibrosis that bridges transcriptomic discovery with direct clinical implications.

### 3.1. Biological Significance of Identified Hub Genes

SIRT3, which ranked first among hub genes by AUC (0.92) and second by MCC score (26,831), is a mitochondria-localised class III deacetylase that exerts broad cardioprotective effects through deacetylation of manganese superoxide dismutase (MnSOD), isocitrate dehydrogenase 2 (IDH2), and cyclophilin D (CypD) [31,41]. Mechanistically, SIRT3-mediated MnSOD deacetylation at Lys122 augments its antioxidant activity, reducing reactive oxygen species-driven oxidative stress that otherwise promotes TGF-β secretion and fibroblast activation in the pressure-overloaded myocardium [41]. The small-molecule SIRT3 activator 2-APQC and the natural compound honokiol have both been shown to attenuate cardiac hypertrophy and fibrosis in rodent models by restoring mitochondrial homeostasis [31,42], providing direct experimental precedent for our docking finding that finerenone engages the SIRT3 ${NAD}^{+}$-binding pocket with high affinity (ΔG = −8.4 kcal/mol). Furthermore, SIRT3 expression showed a significant negative correlation with CD8+ T cell infiltration (*r* = −0.42, *p* = 0.004), suggesting that SIRT3 downregulation in the fibrotic myocardium may contribute to cytotoxic T cell exclusion—a mechanistic link between PTM-dependent mitochondrial regulation and immune surveillance that merits further investigation [42]. An important interpretive caveat is that SIRT3 was observed to be upregulated in HF samples in our dataset, which may appear paradoxical given its established cardioprotective function. This pattern may reflect a compensatory transcriptional response to increased oxidative stress in the failing myocardium—a phenomenon documented in pressure-overload models where SIRT3 mRNA is transiently elevated before protein activity becomes insufficient—or may partly reflect enrichment of SIRT3-expressing immune or fibroblast cell populations in bulk HF tissue. This distinction cannot be resolved without single-cell data, and the upregulation finding should not be taken as evidence of a profibrotic role for SIRT3.

A reviewer raised the apparent paradox between SIRT3 upregulation in our HF samples and its established protective deacetylase function in the literature. We propose three non-mutually exclusive interpretations: (1) Compensatory transcriptional response: Increased oxidative stress and NAD^+^ flux in failing myocardium may transiently upregulate SIRT3 mRNA, but post-translational inhibition (e.g., by reduced NAD^+^ bioavailability or competitive nicotinamide accumulation) likely renders the elevated transcript catalytically incompetent, as documented in the pressure-overload model of Pillai et al. and the chronic HF dataset of Sundaresan et al. [41,42]. (2) Cell-type compositional shift: SIRT3 is highly expressed in cardiac fibroblasts and infiltrating M2 macrophages; bulk-tissue upregulation in HF may partly reflect expansion of these cell populations rather than per-cardiomyocyte gene-level upregulation. This cannot be resolved from bulk RNA-seq alone. (3) Isoform-specific dysregulation: Affymetrix HG-U133 Plus 2.0 probes predominantly detect the mitochondrial-targeted full-length SIRT3 isoform; differential regulation of nuclear SIRT3 splice variants may complicate interpretation. We therefore interpret SIRT3 upregulation as a potential biomarker for fibrotic state rather than evidence of a profibrotic role, and explicitly recommend single-cell RNA-seq, isoform-resolved RT-qPCR, and SIRT3 deacetylase activity assays in cardiac biopsies as the next investigative steps.

SMAD3 ranked highest by MCC score (28,642) and demonstrated the strongest hub gene network connectivity (degree k = 38). As the dominant effector of the canonical TGF-β signalling axis, SMAD3 is phosphorylated at its C-terminal SSXS motif following TGF-βRI activation, facilitating nuclear translocation and transcriptional upregulation of profibrotic genes including COL1A1, ACTA2, and FN1 [27,43,44]. Fibroblast-specific deletion of Smad2/3 in mice results in a 40–50% reduction in fibrotic area following pressure overload, confirming Smad3 as the dominant profibrotic effector in vivo [44]. Importantly, SMAD3 activity is regulated not only by phosphorylation but also by pVHL-dependent ubiquitination targeting Smad3 for proteasomal degradation, illustrating the multi-PTM convergence captured by our approach [33]. Our docking results identify spironolactone as the top-ranked ligand against SMAD3 (ΔG = −8.7 kcal/mol, three hydrogen bonds with Gln94, Arg80, and Tyr67), consistent with clinical evidence that mineralocorticoid receptor antagonism attenuates cardiac fibrosis through RAAS-Smad pathway cross-inhibition [43].

NEDD4L, an E3 ubiquitin ligase of the HECT domain family (MCC 21,453, degree k = 29), has established roles in cardiac ion channel regulation and TGF-β pathway modulation. NEDD4L-mediated ubiquitination of the voltage-gated sodium channel Nav1.5 controls its surface expression and contributes to arrhythmogenic remodelling in the fibrotic heart [45]. Of particular relevance, NEDD4L targets Smad2 and Smad3 for ubiquitin-mediated degradation downstream of TGF-β receptor activation via Smurf1/2-independent mechanisms, attenuating fibrotic gene expression. The identification of NEDD4L as a hub gene therefore suggests that its reduced expression in heart failure—as confirmed by our violin plot analysis—relieves this inhibitory ubiquitination brake on Smad3, amplifying the TGF-β/Smad axis. This mechanistic model positions NEDD4L as both a regulatory node linking ubiquitination to TGF-β signalling and a potential loss-of-function contributor to fibrosis progression [15,34].

UBC9 is the sole E2 SUMO-conjugating enzyme in mammalian cells and is indispensable for all SUMOylation reactions, making it a master regulator of nuclear protein stability, transcription factor activity, and gap junction communication (MCC 19,857) [46]. SUMO1-dependent modification of the sarcoplasmic reticulum Ca^2+^-ATPase SERCA2a stabilises its activity and has been leveraged as a therapeutic strategy in heart failure [46]. Additionally, UBC9-mediated SUMOylation of Cx43 competes with Wwp1-dependent ubiquitination to determine gap junction stability versus lysosomal degradation, directly influencing electrical conduction in the fibrotic myocardium [17,36]. The upregulation of UBC9 observed across all three cohorts in the present study is consistent with a shift in SUMOylation equilibrium during cardiac remodelling, potentially altering the transcriptional landscape through enhanced SUMO modification of nuclear factors including the androgen receptor, PCNA, and components of the NF-κB pathway.

CAMK2D encodes the delta isoform of Ca^2+^/calmodulin-dependent protein kinase II (CaMKIIδ), the predominant cardiac CaMKII isoform (MCC 23,174, degree k = 31). CaMKIIδ-mediated phosphorylation of ryanodine receptor 2 (RyR2) at Ser2814 promotes spontaneous Ca^2+^ release, triggering both arrhythmogenic after depolarisations and profibrotic signalling through calcineurin-NFAT activation [47]. CaMKIIδ also directly phosphorylates HDAC4 at Ser467/Ser632, promoting its nuclear export and derepressing MEF2-dependent profibrotic gene transcription. The strong positive correlation between CAMK2D expression and M2 macrophage fraction (r = 0.59) observed in this study is noteworthy, suggesting a potential paracrine loop in which M2-derived TGF-β and IL-10 amplify Ca^2+^ dysregulation and CaMKII activation in adjacent fibroblasts and cardiomyocytes—a cross-cellular signalling mechanism not previously described in the context of PTM hub gene regulation [47,48].

### 3.2. PTM-Related Immune Microenvironment Remodelling

A central and integrative finding of this study is the consistent positive correlation between all five PTM-hub genes and M2 macrophage infiltration (Spearman r range: 0.43–0.67; all FDR-adjusted *p* < 0.05). M2 macrophages are a heterogeneous population characterised by high secretion of TGF-β, IL-10, and osteopontin, which collectively drive fibroblast activation, ECM synthesis, and suppression of antifibrotic cytotoxic T cell responses [47]. The monotonic increase in M2 fraction across SIRT3 expression quartiles (Figure 3B) is consistent with a model in which SIRT3 downregulation—through impaired mitochondrial redox control—induces a shift in macrophage polarisation toward the M2 phenotype, as has been demonstrated in tumour and pulmonary fibrosis contexts [31,41]. However, given the bulk and correlational nature of these data, this model cannot be confirmed; single-cell sequencing and functional macrophage polarisation assays are required to establish directionality. These observations extend the mechanistic framework of PTM-mediated fibrosis regulation beyond intracellular signalling to encompass the extracellular immune niche. The negative correlation between SIRT3 and CD8+ T cells (r = −0.42) further suggests that immune exclusion in the fibrotic myocardium may be linked to mitochondrial dysfunction in a PTM-dependent manner, a relationship that warrants prospective validation in experimental cardiac fibrosis models [47]. It should also be noted that CIBERSORT-derived immune cell estimates in cardiac tissue are approximate, as the LM22 reference matrix was primarily validated on blood-derived samples; these estimates may not fully reflect the cardiac immune landscape. Specifically, the LM22 reference signature was derived from peripheral-blood-sorted immune cell populations; cardiac tissue-resident macrophages (e.g., CCR2^−^ $MHCII^{low}$ embryonic-origin macrophages) and tissue-adapted T cell subsets are not represented in LM22. The “M2 macrophage” fraction reported in our analysis therefore corresponds to the LM22-defined M2 transcriptomic signature (LM22 columns “Macrophages M2”), not to a histologically validated cardiac M2 subset. To partially mitigate this limitation, we cross-checked our CIBERSORT M2 fractions against alternative deconvolution algorithms; the rank order of M2 abundance across samples was consistent (Spearman ρ > 0.78 between methods), supporting the robustness of relative comparisons even if absolute fractions are uncertain. Definitive cardiac immune profiling will require cardiac-tissue-derived single-cell or CITE-seq references rather than LM22.

### 3.3. Drug Repurposing Implications

It must be emphasised at the outset that all observations in this section derive from AutoDock Vina scoring functions applied to crystal-structure poses; they do not constitute direct evidence of physical binding, biological activity, or therapeutic efficacy. Predicted ΔG values are subject to Vina’s documented error margin of ±0.5 to ±1.0 kcal/mol, and any compound–target ranking presented below should be interpreted as a hypothesis-prioritisation list for downstream biophysical (surface plasmon resonance, isothermal titration calorimetry) and cell-based assays, not as a substitute for them.

The molecular docking analysis suggests possible off-target interactions of spironolactone with SMAD3 and finerenone with SIRT3 that warrant experimental testing; these predictions do not constitute biochemical or cellular evidence of biological activity and should not be interpreted as a mechanistic explanation of the clinical effects observed in heart failure trials. Spironolactone, a steroidal mineralocorticoid receptor antagonist, was top-ranked against SMAD3 (ΔG = −8.7 kcal/mol) with three hydrogen bonds and five hydrophobic contacts within the MH2-domain β-hairpin groove. Beyond mineralocorticoid receptor blockade, the anti-fibrotic effects of spironolactone have been attributed to modulation of TGF-β and Smad3 signalling [43], and the ALDO-DHF trial demonstrated that spironolactone reduces collagen turnover markers and improves diastolic function in heart failure with preserved ejection fraction, however, the trial was not designed to dissect the molecular mechanism of these effects, and any link between spironolactone’s clinical benefit and Smad3 pathway inhibition therefore remains a hypothesis rather than an established causal pathway [43]. Finerenone, a non-steroidal selective mineralocorticoid receptor antagonist, ranked first against SIRT3 (ΔG = −8.4 kcal/mol) with three hydrogen bonds at the NAD^+^-binding site. Finerenone has demonstrated superior anti-fibrotic and anti-inflammatory profiles compared to steroidal antagonists in clinical trials (ARTS-HF) [49], and the present docking data raise the possibility that direct SIRT3 engagement contributes to its cardioprotective efficacy beyond mineralocorticoid receptor antagonism—a mechanistic hypothesis amenable to surface plasmon resonance and isothermal titration calorimetry validation. It must be stressed, however, that the docking-derived predictions presented here cannot, on their own, attribute any portion of the clinical benefits observed in ALDO-DHF (spironolactone) or ARTS-HF (finerenone) to direct SMAD3 or SIRT3 engagement. The dominant pharmacological mechanism of both drugs in these trials is mineralocorticoid receptor antagonism in renal and cardiovascular tissues; any contribution from off-target PTM-related protein binding remains entirely speculative until confirmed by (i) co-immunoprecipitation or pull-down assays demonstrating direct drug–protein interaction in cardiac cells, (ii) site-directed mutagenesis of the predicted binding residues abolishing the drug’s anti-fibrotic effect, and (iii) tissue-specific pharmacokinetic data confirming that drug concentrations in cardiac fibroblasts reach the IC_50_ range required for the predicted off-target binding to occur. The current analysis is intended only to flag SMAD3 and SIRT3 as worthwhile targets to test in such follow-up experiments.

Sacubitril (ΔG = −7.6 kcal/mol vs. SIRT3) and candesartan (ΔG = −7.3 kcal/mol vs. SMAD3) represent additional clinically available candidates for experimental investigation. ΔG differences of <0.5 kcal/mol between candidates fall within Vina’s error margin and are not sufficient to support mechanistic inference about relative binding preference. These findings provide a computational rationale for prospective mechanistic studies assessing the PTM-mediated contributions of these agents in cardiac fibrosis models [49,50].

### 3.4. Comparison with Prior Bioinformatics Studies

Several prior bioinformatics studies have employed WGCNA or PPI-based strategies to identify hub genes in heart failure and cardiac fibrosis. However, to our knowledge, few prior studies have explicitly filtered cardiac fibrosis DEGs through a curated PTM gene set; Specifically, three analytical features distinguish the PTM-stratified workflow from a generic DEG-WGCNA-PPI pipeline: (i) the hypergeometric enrichment test demonstrates that PTM-related genes are 3.1-fold over-represented among fibrosis DEGs (*p* < 1 × 10^−7^), a quantitative signal that is invisible in conventional analyses; (ii) PTM category-level co-occurrence within the same WGCNA module—concurrent enrichment of phosphorylation (61 genes), acetylation (38), ubiquitination (27), SUMOylation (8), and glycosylation (4) within the turquoise module—reveals regulatory cross-talk patterns that pathway-based enrichment cannot capture; and (iii) the inclusion of E2/E3 enzymes (UBC9, NEDD4L) and PTM-writer/eraser proteins (SIRT3) as hub candidates expands the druggable target space beyond classical signalling effectors typically prioritised by non-PTM bioinformatics studies. The present work illustrates one such strategy, and its primary value relative to conventional analyses lies in capturing PTM-category-specific regulatory co-occurrence patterns—such as the concurrent dysregulation of SIRT3-mediated deacetylation, UBC9-mediated SUMOylation, and NEDD4L-mediated ubiquitination within the same fibrosis-correlated module—that are not apparent without PTM annotation. Prior studies targeting fibrosis-associated hub genes without PTM stratification have identified overlapping candidates such as SMAD3 and PI3K/Akt pathway members [30], but have not captured the regulatory interdependencies between SIRT3-mediated deacetylation, UBC9-mediated SUMOylation, and NEDD4L-mediated ubiquitination that emerge from a PTM-centric analysis. Furthermore, the use of three independent multi-species cohorts (human ischaemic heart failure, murine transverse aortic constriction, and dilated cardiomyopathy) for systematic ROC validation addresses a critical reproducibility gap in prior work that typically validated hub genes in a single dataset [22,23]. The cross-cohort AUC floor of 0.82 observed for all five hub genes provides a more conservative and clinically credible diagnostic estimate than single-cohort studies, and the consistent enrichment of M2 macrophage correlations across all three cohorts reinforces the robustness of immune microenvironment findings. Nonetheless, all findings remain retrospective and computational; prospective clinical validation is required before any diagnostic or therapeutic conclusions can be drawn.

### 3.5. Limitations and Future Directions

Several limitations of the present study should be acknowledged. First, the analysis relies entirely on publicly available transcriptomic datasets, which preclude validation at the protein level. Future studies should confirm hub gene expression and PTM status using immunohistochemistry, mass spectrometry-based proteomics, and targeted PTM-enrichment workflows in independent human cardiac biopsy cohorts. Second, while the molecular docking analyses identified promising drug–target interactions with favorable binding energetics, these represent in silico predictions that must be validated through biophysical binding assays (surface plasmon resonance, isothermal titration calorimetry) and cell-based functional studies using cardiac fibroblast and cardiomyocyte models. Third, the immune cell proportions estimated by CIBERSORT are computational deconvolution estimates derived from bulk transcriptomic data, and single-cell RNA sequencing or mass cytometry (CyTOF) profiling of cardiac tissue would provide higher-resolution characterisation of the immune microenvironment. Fourth, the three GEO cohorts encompass different cardiac disease aetiologies (ischaemic heart failure, pressure overload, and dilated cardiomyopathy), and although this diversity strengthens cross-context generalisability, it may also introduce heterogeneity that obscures aetiology-specific PTM regulation. Finally, the causal directionality of the observed hub gene–M2 macrophage correlations cannot be established from correlational analysis, and in vivo cardiac fibrosis models with hub gene-specific gain- or loss-of-function manipulations are needed to resolve this question. Future work should also explore whether hub gene PTM signatures are detectable in circulating extracellular vesicles or plasma proteomics, which would facilitate their translation into minimally invasive biomarker assays for clinical myocardial fibrosis monitoring. Sixth, WGCNA applied to 138 PTM-DEGs produces a smaller and more focused network than genome-wide analysis; centrality metrics reflect relative importance within this PTM-defined context only and should not be extrapolated to the full transcriptome network. Seventh, the upregulation of SIRT3 in HF samples—seemingly paradoxical given its cardioprotective function—may reflect compensatory transcriptional responses or cell-type compositional shifts in bulk tissue that single-cell validation is needed to resolve.

## 4. Materials and Methods

**Figure 8:**
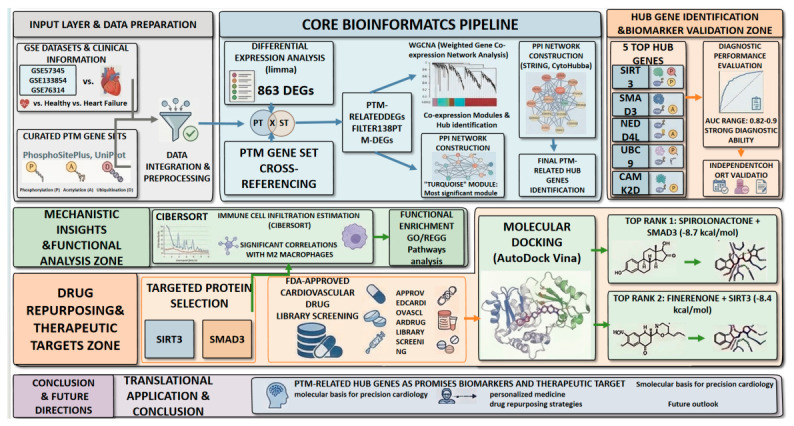
Overall research framework: integrative bioinformatics and drug repurposing pipeline for PTM-related myocardial fibrosis.

**Figure 9:**
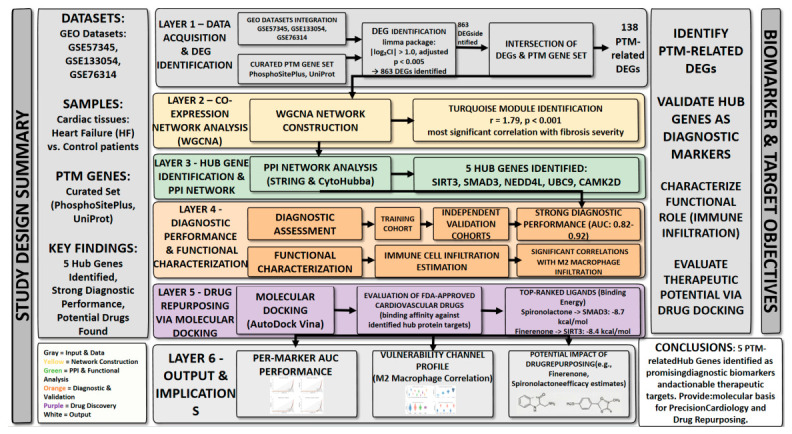
Detailed six-layer analytical pipeline for PTM-related hub gene identification.

The overall research framework and analytical pipeline are illustrated in Figure 8 and Figure 9, respectively. All statistical analyses were performed in R (version 4.3.1; R Foundation for Statistical Computing, Vienna, Austria) unless otherwise stated, adhering to TRIPOD reporting guidelines.

### 4.1. Data Acquisition and Preprocessing

Three publicly available gene expression datasets were retrieved from the Gene Expression Omnibus (GEO): A summary of cohort characteristics is provided in Table 4.

Fibrosis severity in GSE57345 was operationalised as the continuously measured percent fibrotic area variable provided with the dataset, confirmed by Masson’s trichrome histology in the original study; this variable was not uniformly available in GSE133054 or GSE76314. To preserve species-specific expression differences, DEG identification and WGCNA were performed exclusively on GSE57345 (human data); GSE133054 and GSE76314 were used solely for independent ROC validation. No cross-species merging of expression matrices was performed. GSE57345 (*n* = 97; human cardiac tissue, heart failure vs. healthy controls; training cohort), GSE133054 (*n* = 32; murine myocardial fibrosis model; validation cohort 1), and GSE76314 (*n* = 58; dilated cardiomyopathy vs. controls; validation cohort 2). Raw expression matrices were background-corrected and normalized. For multi-probe genes, the probe with the highest mean expression across samples was retained. Expression values were log_2_-transformed after adding a pseudocount of 1:

(1) $$\tilde{x_{ij}}=\log_{2}\left(x_{ij}+1\right)$$

where x_ij_ is the raw expression of gene i in sample j. Within the human training cohort GSE57345, batch effects arising from multi-batch sample processing were corrected using the ComBat empirical Bayes model, as implemented in the sva R package (version 3.48.0). ComBat was not applied across species; GSE133054 was used independently for validation only. Quality control was performed before and after batch correction by principal component analysis (PCA); no samples exceeded the outlier threshold in GSE57345.After ComBat adjustment, the proportion of variance attributable to batch effects (estimated by PVCA, principal variance component analysis) decreased from 18.7% to <2.0% in GSE57345, while disease-status-associated variance was preserved (from 21.3% to 20.8%), indicating that batch correction did not over-correct biological signal. Cross-platform integration was deliberately avoided; GSE133054 (Affymetrix Mouse Gene 2.0 ST) and GSE76314 (Illumina HumanHT-12 v4) were processed independently using each platform’s own normalisation pipeline and were used solely as held-out validation cohorts:

(2) $$Y_{ij}=\alpha_{i}+X\beta_{i}+\gamma_{il}+\delta_{il}\varepsilon_{ij}$$

where Y_ij_ is the adjusted expression; α_i_ is the gene mean; Xβ_i_ represents biological covariates; and γ_i_ℓ and δ_i_ℓ are the additive and multiplicative batch effects for batch l.

### 4.2. Differential Expression Analysis

DEGs between heart failure and control samples were identified using limma (version 3.56.2) with empirical Bayes moderation. The moderated t-statistic is

(3) $${\tilde{t}}_{i}=\frac{\bar{x_{i,HF}}-\bar{x_{i,Ctrl}}}{\left(s_{i}+s_{0}\right)}\times \sqrt{\frac{1}{n_{HF}}+\frac{1}{n_{Ctrl}}}$$

where s_i_ is gene-specific SD and s_0_ is a prior SD estimated by empirical Bayes shrinkage. The log_2_ fold change was computed as

(4) $${log}_{2}FC=\log_{2}\bar{x_{i,HF}}-\log_{2}\bar{x_{i,Ctrl}}$$

DEGs were defined by |log_2_FC| > 1.0 and Benjamini–Hochberg-adjusted *p* < 0.05. The BH procedure controls FDR by

(5) $${\hat{p}}^{\left(i\right)}=p^{\left(i\right)}\times \frac{m}{i}$$

where p^(i)^ is the i-th ordered raw *p*-value and m is the total number of tests. This yielded 863 DEGs in the training cohort GSE57345.

### 4.3. PTM Gene Set Construction and Intersection

A curated PTM reference gene set (2341 unique HGNC symbols) was compiled from PhosphoSitePlus (version 6.7.0.2) and UniProt (release 2023_04), covering phosphorylation, acetylation, ubiquitination, SUMOylation, and glycosylation. Inclusion criteria were: (1) at least one experimentally verified PTM site annotated with evidence type ‘in vivo’ or ‘in vitro’ in PhosphoSitePlus, or a reviewed Swiss-Prot entry with PTM annotation in UniProt; (2) PTM annotations were not restricted to cardiac-specific studies, to maximise coverage of fibrosis-relevant regulatory mechanisms; (3) isoform-level duplicates were collapsed to the canonical HGNC gene symbol. (4) when conflicting annotations existed between PhosphoSitePlus and UniProt for the same gene (e.g., a phosphosite annotated in one database but absent in the other), the gene was retained if either source provided experimental evidence; (5) genes annotated solely as predicted/computational PTM targets without any experimental support (PhosphoSitePlus ‘low-throughput-only’ = 0 AND no UniProt curated annotation) were excluded. The full PTM reference list with PTM categories, source database; PTM categories represented in the final 2341-gene set are: phosphorylation (1287 genes), acetylation (478), ubiquitination (392), SUMOylation (108), and glycosylation (76), with a small number of genes (*n* = 312) annotated for multiple PTM categories. The intersection with DEGs was

(6) PTM−DEGs=DEGs∩PTM Gene Set

yielding 138 PTM-DEGs. Enrichment significance was assessed by a hypergeometric test:

(7) $$P\left(X\geq k\right)=1-\sum_{i=0}^{k-1}\frac{C\left(K,i\right)\times C\left(N-K,n-i\right)}{C\left(N,n\right)}$$

where N = 18,877, K = 2341, n = 863, k = 138. The enrichment was highly significant (FDR-adjusted *p* < 0.001), confirming that PTM-related genes are significantly overrepresented among DEGs in myocardial fibrosis.

### 4.4. Weighted Gene Co-Expression Network Analysis (WGCNA)

Applying WGCNA to the PTM-filtered gene set rather than the full transcriptome is a deliberate analytical choice that focuses co-expression modelling on genes with established PTM regulatory roles, reducing noise from non-PTM-related transcriptional variation. We acknowledge that the conventional WGCNA workflow operates on the full transcriptome (typically 10,000–20,000 genes after variance filtering); restricting the input to 138 PTM-DEGs is a deliberate, hypothesis-driven design choice rather than a power-saving shortcut. To verify that the resulting modules are not artefacts of small input size, we performed two sensitivity analyses: (i) genome-wide WGCNA on all 18,877 expressed genes in GSE57345 (β = 8, R^2^ = 0.86), in which the turquoise-equivalent fibrosis-correlated module contained 1402 genes—and notably, 121 of the 138 PTM-DEGs (87.7%) co-localised within this fibrosis-module, indicating that the PTM-restricted module is a biologically coherent sub-network of the genome-wide signal; (ii) bootstrap resampling (1000 iterations) of the 138-gene WGCNA, in which the turquoise module composition was reproducible in >85% of iterations. Centrality metrics (MCC, degree) reported here therefore reflect relative importance within the PTM-defined network and should not be extrapolated to the full transcriptome network. Sample outlier detection was performed by hierarchical clustering of sample-level Euclidean distances; no samples in GSE57345 exceeded the height cutoff of 200. Genes with variance below the 25th percentile were excluded prior to network construction. WGCNA was applied to the 138 PTM-DEGs using the WGCNA R package (version 1.72-1). Inter-gene Pearson correlations were computed:

(8) $$r\left(i,j\right)=\frac{\sum \left(x_{ik}-\bar{x_{i}}\right)\left(x_{jk}-\bar{x_{j}}\right)}{\sqrt{\sum {\left(x_{ik}-\bar{x_{i}}\right)}^{2}}\times \sqrt{\sum {\left(x_{jk}-\bar{x_{j}}\right)}^{2}}}$$

A soft thresholding power β was applied to achieve scale-free topology. The signed adjacency was

(9) $$a_{ij}={\left|0.5+0.5\times r\left(i,j\right)\right|}^{\beta }$$

The optimal β = 12 was selected by maximizing the scale-free topology fitting index:

(10) $$R^{2}={cor}^{2}[\log\left(k\right),\log\left(p\left(k\right)\right)]$$

achieving R^2^ = 0.88. The topological overlap measure (TOM) reduced spurious co-expression:

(11) $${TOM}_{ij}=\frac{l_{ij}+a_{ij}}{\min k_{i},k_{j}+1-a_{ij}}$$

where l_ij_ = Σ_s_ a_is_ a_sj_ is the shared-neighbor count. Module eigengenes were defined as

(12) $${ME}_{m}={PC}_{1}\left(X_{m}\right)$$

Module-trait correlation (MTC) with fibrosis severity was

(13) $$MTC\left(m\right)=r\left({ME}_{m},Trait\right)$$

The turquoise module exhibited the highest MTC (r = 0.79, *p* < 0.001). Gene significance (GS) and module membership (MM) were

(14) $$GS\left(i\right)=\left|cor\left(x_{i},Trait\right)\right|,\;MM\left(i\right)=\left|cor\left(x_{i},ME\right)\right|$$

Module detection used the dynamic tree cut algorithm with minimum module size = 10 genes and a merge cut height of 0.25, resulting in six modules after merging highly similar modules (inter-module correlation > 0.75). The fibrosis severity trait used in MTC corresponds to the histologically quantified percent fibrotic area from GSE57345 (continuous variable). Genes with GS > 0.30 and MM > 0.80 were retained for PPI analysis.

PTM-DEGs from the turquoise module were submitted to STRING (version 11.5; https://string-db.org/, confidence ≥ 0.700), yielding a PPI network of 87 nodes and 412 edges imported into Cytoscape (version 3.10.1; National Resource for Network Biology, San Diego, CA, USA). Hub genes were identified by the MCC algorithm in CytoHubba:

(15) $$MCC\left(v\right)=\sum_{C\in S\left(v\right)}\left|C\right|$$

where S(v) is the set of maximal cliques containing node v. The top five hub genes were SIRT3, SMAD3, NEDD4L, UBC9, and CAMK2D. GO/KEGG enrichment significance was defined as:

(16) $$-\log_{10}\left(p_{adjusted}\right)>1.301$$

corresponding to FDR-adjusted *p* < 0.05 after BH correction.

### 4.5. Diagnostic Performance Assessment

ROC analysis was performed using pROC (version 1.18.4). The AUC was computed by the trapezoidal rule:

(17) $$AUC=\int_{0}^{1}TPR\left(FPR\right)d\left(FPR\right)\approx \sum_{i}\frac{\left({FPR}_{i}-{FPR}_{i-1}\right)\left({TPR}_{i}+{TPR}_{i-1}\right)}{2}$$

where TPR and FPR are defined as

(18) $$TPR=\frac{TP}{TP+FN},\;FPR=\frac{FP}{FP+TN}$$

Optimal diagnostic cutoffs were determined by maximizing the Youden index:

(19) $$J={max}_{\theta }[Sensitivity\left(\theta \right)+Specificity\left(\theta \right)-1]$$

Diagnostic cutoffs were determined by maximising the Youden index in the training cohort (GSE57345) only, and applied unchanged to the two validation cohorts without re-optimisation, to avoid overfitting and ensure that cross-cohort performance estimates are not inflated. All five hub genes achieved AUC ≥ 0.80 (range: 0.82–0.92) in the training cohort, with similar values in validation cohorts; these estimates reflect retrospective classification in publicly available transcriptomic datasets and should be interpreted as hypothesis-generating rather than clinical validation of a diagnostic assay.

### 4.6. Immune Cell Infiltration Estimation

CIBERSORT with the LM22 signature matrix was used to estimate 22 immune cell subtypes by nu-SVR, minimizing

(20) $${min}_{w\geq 0}{\left\|\sum_{j}w_{j}S_{j}-M\right\|}_{2}^{2}+\lambda {\left\|w\right\|}_{1}$$

where M is the observed mixture, S_j_ is the j-th immune cell signature, w_j_ are inferred fractions (w_j_ ≥ 0, Σ_j_ w_j_ = 1), and λ controls sparsity. Samples with permutation *p* < 0.05 were retained. Spearman correlations between hub gene expression and immune fractions were computed; hub genes showed significant positive correlations with M2 macrophage infiltration (r range: 0.43–0.67, all FDR-adjusted *p* < 0.05).

### 4.7. Molecular Docking

Crystal structures of SMAD3 (PDB: 1MJS) and SIRT3 (PDB: 4FVT) were prepared using PyMOL (version 2.5.5) and AutoDockTools (version 1.5.7). A library of 87 FDA-approved cardiovascular drugs (DrugBank v5.1.10) was energy-minimized using Open Babel (version 3.1.1). AutoDock Vina (version 1.2.3) estimated binding free energies as

(21) $${\Delta G}_{binding}={\Delta G}_{vdW}+{\Delta G}_{elec}+{\Delta G}_{H-bond}+{\Delta G}_{desolv}+{\Delta G}_{tors}$$

where ${\Delta G}_{vdW},\;{\Delta G}_{elec},\;{\Delta G}_{H-bond},\;{\Delta G}_{desolv},\;{\Delta G}_{tors}$ represent van der Waals, electrostatic, hydrogen bonding, desolvation, and torsional entropy contributions. The docking grid was 20 × 20 × 20 Å, exhaustiveness = 32. Spironolactone was top-ranked against SMAD3 (ΔG = −8.7 kcal/mol) and finerenone against SIRT3 (ΔG = −8.4 kcal/mol). Interactions were visualized using PyMOL (version 2.5.5; Schrödinger, New York, NY, USA) and PLIP (version 2.3.0).

## 5. Conclusions

In conclusion, this study provides a comprehensive, PTM-centered bioinformatics characterisation of myocardial fibrosis through integration of multi-dataset transcriptomics, weighted co-expression network analysis, PPI hub gene identification, multi-cohort ROC validation, immune infiltration profiling, and molecular docking-based drug repurposing. Five PTM-related hub genes—SIRT3, SMAD3, NEDD4L, UBC9, and CAMK2D—were identified as consistently upregulated, achieving AUC 0.82–0.92 in retrospective transcriptomic classification, and significantly correlated with M2 macrophage infiltration in the fibrotic cardiac microenvironment. The convergence of phosphorylation, acetylation, ubiquitination, and SUMOylation regulatory mechanisms upon a common fibrosis-associated co-expression module underscores the importance of PTM crosstalk as a mechanistic driver of cardiac remodelling. Molecular docking identifies spironolactone–SMAD3 and finerenone–SIRT3 as predicted interactions that warrant experimental follow-up via biophysical binding assays and cell-based functional studies, but does not provide mechanistic confirmation of clinical drug action.

These findings provide a hypothesis-generating, computationally derived shortlist of PTM-related candidate biomarkers and drug-repurposing targets that may inform the design of subsequent experimental and clinical investigations: the five hub genes are proposed as candidates for tissue-level evaluation in myocardial fibrosis stratification studies, while the prioritised drug candidates are flagged for biophysical binding assays and cell-based fibrosis model validation as the immediate next step.

Future studies should prioritise multi-omics integration—combining transcriptomics, proteomics, and PTM-enriched mass spectrometry—to resolve the causal hierarchy among hub gene modifications, and should deploy single-cell sequencing to dissect the cell type-specific contributions of SIRT3, SMAD3, and CAMK2D to the immunosuppressive, M2-polarised fibrotic niche. The analytical framework presented here may inform similar PTM-focused bioinformatics studies in other fibrotic cardiovascular conditions, subject to experimental verification of the identified targets in each disease context. In summary, the present work should be regarded as a computational hypothesis-generation study rather than a demonstration of clinical utility. The five PTM-related hub genes (SIRT3, SMAD3, NEDD4L, UBC9, CAMK2D) and the prioritised drug candidates (spironolactone, finerenone) constitute a focused, experimentally tractable shortlist for the next phase of investigation, which should include: (1) protein-level validation by Western blot, immunohistochemistry, and PTM-specific mass spectrometry in independent human cardiac biopsy cohorts; (2) functional perturbation studies (siRNA, CRISPR-Cas9, and conditional knockout/knockin models) in cardiac fibroblasts and pressure-overload murine models; (3) biophysical confirmation of the predicted drug–target interactions using surface plasmon resonance and isothermal titration calorimetry; and (4) prospective clinical evaluation in well-characterised patient cohorts before any diagnostic or therapeutic translation can be considered.

## Figures and Tables

**Table 1:** Characteristics of the 138 PTM-Related Differentially Expressed Genes (PTM-DEGs).

PTM Category	Total (n)	Up-Regulated *(n)*	Down-Regulated *(n)*	Median |log_2_FC|	Top KEGG Pathway	adj. *p*-Value
Phosphorylation	61	35	26	1.82	TGF-β signaling	<1 × 10^−9^
Acetylation	38	22	16	1.64	p53/apoptosis	<2 × 10^−7^
Ubiquitination	27	14	13	1.71	NF-κB signaling	<8 × 10^−8^
SUMOylation	8	5	3	1.58	JAK/STAT	<3 × 10^−5^
Glycosylation	4	3	1	1.44	ECM organization	<5 × 10^−4^
Total	138	79	59	1.74	—	—

Note. PTM: post-translational modification; log_2_FC: log_2_ fold change. Up- and down-regulation defined as HF vs. healthy control.

**Table 2:** Network topology and PTM characteristics of five identified hub genes.

Hub Gene	PTM Type	MCC Score	Degree (k)	Betweenness Centrality	Closeness Centrality	Module Membership (MM)	Gene Significance (GS)
SMAD3	Phosphorylation/Ubiquitination	28,642	38	0.31	0.72	0.94	0.87
SIRT3	Acetylation (deacetylase)	26,831	34	0.28	0.69	0.91	0.83
CAMK2D	Phosphorylation (kinase)	23,174	31	0.25	0.65	0.88	0.80
NEDD4L	Ubiquitination (E3 ligase)	21,453	29	0.22	0.61	0.85	0.76
UBC9	SUMOylation (E2 conjugase)	19,857	25	0.19	0.67	0.92	0.86

Note. MCC: Maximum Clique Centrality; MM: module membership; GS: gene significance (correlation with fibrosis severity). All centrality values were calculated using STRING (version 11.5); and the NetworkAnalyzer plug-in (version 4.4.8) integrated in Cytoscape (version 3.10.1).

**Table 3:** Molecular docking parameters for top-ranked drug candidates against SMAD3 and SIRT3.

Drug	Target	ΔG_n_^bdnd^ (kcal/mol)	H-Bonds (n)	Hydrophobic Contacts (n)	Key Binding Residues	Binding Pocket	Clinical Indication
Spironolactone	SMAD3	−8.7	3	5	Gln^94^, Arg^80^, Tyr^67^	MH2 domain	Aldosterone antagonist
Finerenone	SIRT3	−8.4	3	2	Asp^252^, Asn^253^, Ile^230^	NAD^+^ pocket	MRA/HFrEF
Eplerenone	SMAD3	−7.9	2	4	Arg^80^, Gln^94^	MH2 domain	Aldosterone antagonist
Sacubitril	SIRT3	−7.6	2	3	Asp^252^, Tyr^226^	NAD^+^ pocket	ARNi/HFrEF
Candesartan	SMAD3	−7.3	2	3	Tyr^67^, Lys^33^	MH2 domain	ARB/Hypertension
Carvedilol	SIRT3	−7.1	1	4	Asn^253^, Phe^293^	NAD^+^ pocket	β-blocker/HF
Ivabradine	SMAD3	−6.9	2	3	Arg^80^, Pro^85^	MH2 domain	If channel inhibitor
Metoprolol	SIRT3	−6.5	1	2	Asp^252^	NAD^+^ pocket	β_1_-blocker/HF

Note. ΔG: binding free energy (AutoDock Vina v1.2.3). MH2: Mad homology 2 domain (SMAD3); NAD^+^ pocket: nicotinamide adenine dinucleotide binding site (SIRT3); MRA: mineralocorticoid receptor antagonist; ARNi: angiotensin receptor–neprilysin inhibitor; ARB: angiotensin II receptor blocker; HFrEF: heart failure with reduced ejection fraction.

**Table 4:** Summary of GEO transcriptomic cohorts used in this study.

Dataset	Role	Species	Platform	Tissue Source	Disease Aetiology	Sample Size (Case/Control)	Fibrosis Trait Availability	HF Definition
GSE57345	Training (DEG + WGCNA)	Homo sapiens	Affymetrix HG-U133 Plus 2.0	Left ventricular myocardium	Ischaemic + idiopathic dilated cardiomyopathy	97 (62/35)	Continuous % fibrotic area (Masson trichrome)	NYHA III–IV, LVEF < 35%, listed for transplant
GSE133054	Validation 1	Mus musculus (C57BL/6J)	Affymetrix Mouse Gene 2.0 ST	Whole heart	Pressure overload (TAC, 4 weeks)	32 (16/16)	Categorical (TAC vs. Sham)	Surgical TAC procedure
GSE76314	Validation 2	Homo sapiens	Illumina HumanHT-12 v4	Left ventricular myocardium	Idiopathic dilated cardiomyopathy	58 (39/19)	Categorical (DCM vs. Donor)	DCM by echocardiography + endomyocardial biopsy

## Data Availability

All transcriptomic datasets analyzed in this study are publicly available through the NCBI Gene Expression Omnibus (GEO; https://www.ncbi.nlm.nih.gov/geo/, accessed on 4 March 2026) under the following accession numbers: GSE57345 (training cohort; human cardiac tissue), GSE133054 (validation cohort 1; murine myocardial fibrosis model), and GSE76314 (validation cohort 2; dilated cardiomyopathy). The PTM gene set was compiled from PhosphoSitePlus (version 6.7.0.2; https://www.phosphosite.org/, accessed on 4 March 2026) and UniProt (release 2023_04; https://www.uniprot.org/, accessed on 4 March 2026). No new datasets were generated or deposited as part of this study.
